# Bactericidal Antibiotics Increase Hydroxyphenyl Fluorescein Signal by Altering Cell Morphology

**DOI:** 10.1371/journal.pone.0092231

**Published:** 2014-03-19

**Authors:** Wilhelm Paulander, Ying Wang, Anders Folkesson, Godefroid Charbon, Anders Løbner-Olesen, Hanne Ingmer

**Affiliations:** 1 Department of Veterinary Disease Biology, University of Copenhagen, Frederiksberg C, Denmark; 2 Department of Microbial Ecology and Animal health, Technical University of Denmark, Frederiksberg C, Denmark; 3 Department of Biology, University of Copenhagen, Copenhagen, Denmark; University of Massachusetts Medical School, United States of America

## Abstract

It was recently proposed that for bactericidal antibiotics a common killing mechanism contributes to lethality involving indirect stimulation of hydroxyl radical (OH^•^) formation. Flow cytometric detection of OH^•^ by hydroxyphenyl fluorescein (HPF) probe oxidation was used to support this hypothesis. Here we show that increased HPF signals in antibiotics-exposed bacterial cells are explained by fluorescence associated with increased cell size, and do not reflect reactive oxygen species (ROS) concentration. Independently of antibiotics, increased fluorescence was seen for elongated cells expressing the oxidative insensitive green fluorescent protein (GFP). Although our data question the role of ROS in lethality of antibiotics other research approaches point to important interplays between basic bacterial metabolism and antibiotic susceptibility. To underpin such relationships, methods for detecting bacterial metabolites at a cellular level are needed.

## Introduction

Most clinically used antibiotics target key cellular processes such as cell-wall assembly, protein synthesis, or DNA replication in order to kill (bactericidal) or inhibit growth of (bacteriostatic) bacterial cells. However, it was recently proposed that three classes of bactericidal antibiotics with unrelated primary targets induce the production of highly reactive oxygen species (ROS) such as hydroxyl radicals (OH^•^) and that these molecules contribute to the killing of bacterial cells. According to this hypothesis, the bactericidal antibiotics, via the tricarboxylic acid cycle, cause an over stimulation of the electron transport chain and release of iron from the iron-sulfur clusters that activates the Fenton reaction, resulting in terminal damage to bacterial DNA, protein and lipids [1]–[4].

Evidence offered to support this hypothesis included the observation of increased oxidation of cell-penetrating hydroxyphenyl fluorescein (HPF) dye in antibiotics-treated bacteria as measured by flow cytometry [1]. Cell-penetrating HPF dye exhibits low fluorescence when inactive but strong, dose-dependent fluorescence when oxidized by highly reactive oxygen species such as hydroxyl radicals and peroxynitrite [5]. Elevated signals from HPF-dyed cells can thus in principle be used to demonstrate increased ROS activity.

In recent experiments, elevated HPF signals from bacterial cells exposed to lethal concentrations of kanamycin, ampicillin and norfloxacin were interpreted as evidence of ROS formation in response to the antibiotics [1]. Subsequent reports have disputed the ROS contribution to the killing effect of bactericidal antibiotics [6]–[8]. The supporting data include results showing that bactericidal antibiotics neither increase the rate of hydrogen peroxide formation, nor elevate the levels of intracellular iron necessary for the Fenton reaction to proceed. Importantly, the antibiotic lethality also persisted in the absence of oxygen [7], [8]. These reports did however not explain why HPF signals were elevated in antibiotic-treated cells.

Here, we show that exposure to bactericidal antibiotics such as norfloxacin increases bacterial cell size as measured by forward scatter (FSC) in the flow cytometer. We also demonstrate that larger cells yield higher fluorescence signals, whether derived from the HPF probe or from constitutively expressed green fluorescent protein (GFP), which signal is independent of the ROS concentration. Moreover, a clear correlation between cell size and fluorescence is detected in the absence of antibiotics for the *ftsI*2158 temperature sensitive cell division mutant that at non-permissive temperature forms filaments with increased size.

## Results

### Effect of antibiotics on cell size

**Figure 1:**
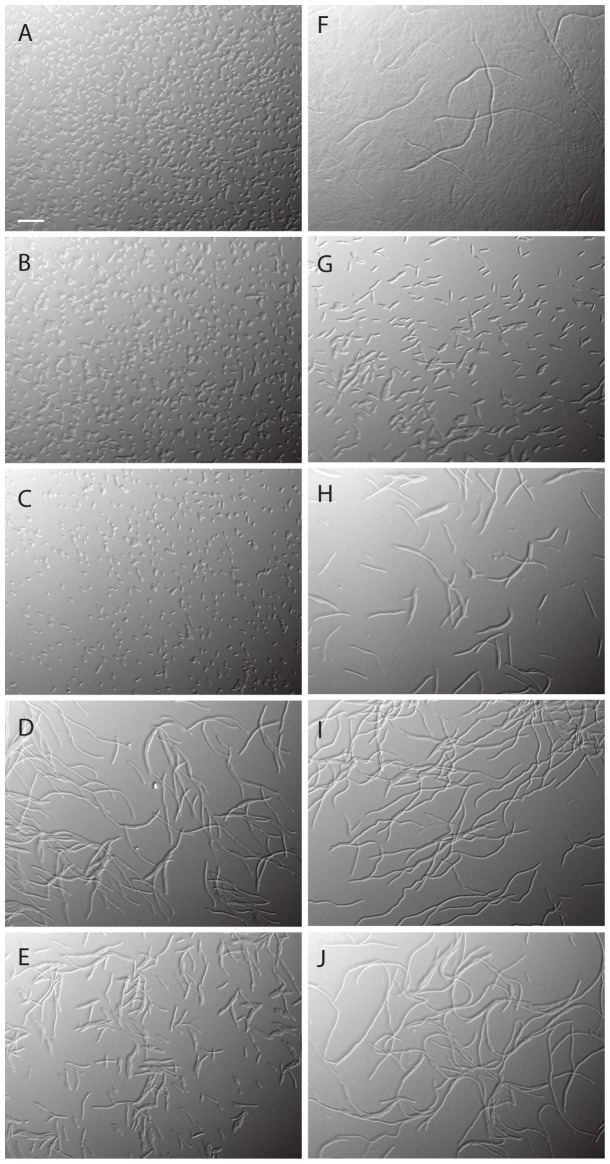
Differential interference contrast (DIC) micrographs of wild type *E. coli* MG1655, untreated (A), treated for 3 hours with kanamycin 5 μg/ml (B), norfloxacin 25 ng/ml (C), norfloxacin 250 ng/ml (D), norfloxacin 2500 ng/ml (E), 3 hours to ampicillin 5 μg/ml (F) or *ftsI*2158 Grown at 30°C (G), 1 hour at 42°C (H) 2 hours at 42°C (I) 3 hours at 42°C (J). The magnification is the same for all panels. Scale bar is 120 μm.

To assess the impact of ampicillin (5 μg/ml), norfloxacin (25 ng/ml, 250 ng/ml and 2500 ng/ml) and kanamycin (5 μg/ml) on morphology of wild-type *E. coli* MG1655 cells, differential interference contrast (DIC) microscopy was used. Antibiotic concentrations were selected based on their bactericidal potential in previous experiments [1]. Samples were examined by microscopy prior to and 3 hours post antibiotic treatment. For the cells exposed to ampicillin and 250 ng/ml norfloxacin an on average 9.4 fold increase in relative cell size compared to the three hours untreated control was observed (see figure 1 D and F and Table S1). For the kanamycin treated cells the increase in relative cell size was 1.6 fold (see figure 1 D and Table S1).

### Impact of cell size on HPF fluorescence

**Figure 2:**
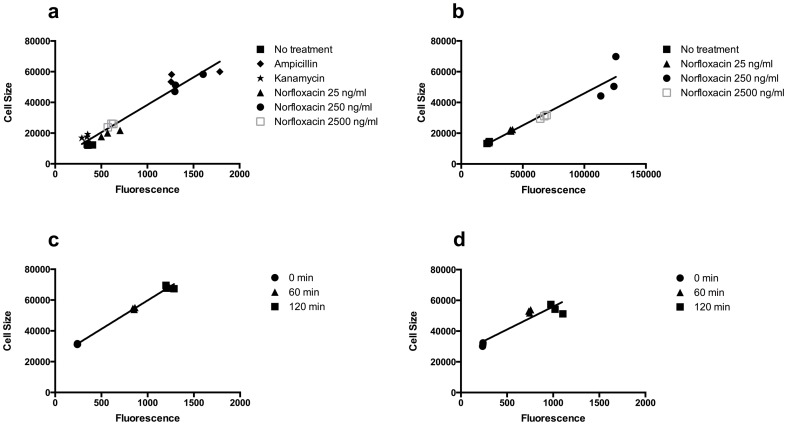
Correlation between average fluorescent HPF/GFP (FL1-A) signal and average cell size (FSC-A) as measured by flow cytometry for a) MG1655 cells incubated with HPF probe with or without antibiotics as indicated, b) SAR18 cells expressing chromosomally encoded GFP incubated either without or with a 25 ng/ml, 250 ng/ml or 2500 ng/ml concentration of norfloxacin, cells expressing the *ftsI*2158 temperature sensitive cells incubated in growth medium at 42°C and monitored at the indicated time points with HPF c) or with out HPF present d). (See log FL1-A vs log FSC-A data plotted in Figure S1).

To study the relationship between cell morphology and fluorescence, two fluorescent markers were employed: a cell-penetrating HPF probe used for detecting ROS and a chromosomally encoded GFP, the activity of which does not vary with oxidation [9]. In cells incubated with the HPF probe and exposed to ampicillin and norfloxacin (25 ng/ml, 250 ng/ml or 2500 ng/ml), average cell size (measured as FSC) and HPF fluorescence (measured as FL-1 signal) correlated linearly (figure 2a). Similar results were obtained with cells analyzed at two different flow rates (data not shown). In contrast, kanamycin only marginally affected cell size and HPF fluorescence at the concentrations applied (R-square = 0.94; figure 2a). In cells constitutively expressing GFP and exposed to norfloxacin (25 ng/ml, 250 ng/ml and 2500 ng/ml), average cell size and GFP fluorescence also correlated linearly with a decrease in cell size and GPF fluorescence observed for the highest norfloxacin concentration (R-square = 0.91; figure 2b).

Subsequently we monitored the relationship between cell size and HPF fluorescence in cells without antibiotic exposure using the temperature sensitive *ftsI*2158 cell division mutant. The *ftsI*2158 mutation results in a thermo-labile Penicillin Binding Protein 3 (PBP3), the septation specific peptidoglycan transpeptidase, [10]. When shifted from 30°C to 42°C, cell division is inhibited and *ftsI*2158 cells undergo extensive filamentation (figure 1 G to K). Samples were analyzed by flow cytometry at 0, 60 and 120 min after temperature shift in the presence of the HPF probe. Importantly, average cell size and HPF fluorescence correlated linearly (R-square = 0.99; figure 2c). To address if auto-fluorescence contributes to the monitored signal, we examined the *ftsI*2158 mutant at 42°C in the absence of HPF. Again we could establish a correlation between cell size and fluorescent signal (R-square = 0.85; figure 2d). Thus, cell size appears to be the main contributor to the increased fluorescence signal.

## Discussion

Flow-cytometric analysis of HPF probe fluorescence has been used to show that bactericidal antibiotics induce the production of ROS such as hydroxyl radicals. This observation led to the proposal that reactive oxygen molecules play a role in the killing effect of bactericidal antibiotics [1]. Here we show that elevated HPF signal primarily stems from antibiotics causing increased cell size (figure 2a) rather than ROS-mediated HPF oxidation. The relationship between cell size and fluorescent signal shift is supported by the findings that: i) cells carrying a chromosomally located GFP marker, which is insensitive to oxidation, showed a correlation between increase in cell size and fluorescent signal when exposed to norfloxacin (figure 2b), ii) for the temperature sensitive cell division mutant *ftsI*2158 cell size and fluorescence signal corelated when shifted to non-permissive temperature in the absence of antibiotics (figure 2c), iii) cells treated with kanamycin showed no significant changes in cell size or HPF signal (figure 2a). The lack of HPF signal in the presence of kanamycin concords with observations made by Keren *et al*., [7] and disagrees with those of Kohanski *et al*., [1]. The reason for this inconsistency is not clear. Furthermore, we found increased flow cytometric signal for elongated *ftsI*2158 mutant cells even in the absence of HPF probe (figure 2d), which suggests that the flow cytometric measurements not only record the HPF signal but also additional fluorescence. Just recently, autofluorescence has been put forward as a potential explanation to this observation and our data supports that this may be the case (figure 2d) [11].

Filamentation, in which cells continue to elongate but do not divide, is often observed in bacteria exposed to various stresses. Filamentation of *E. coli* in response to beta-lactams [12] and fluoroquinolones [12], [13] is well known. The effect of fluoroquinolones has been shown to be dose dependent with greater filamentation at the minimal inhibitory concentration (MIC) of ciprofloxacin compared to 100 times the MIC [13]. We found a significantly reduced elongation and decreased HPF/GFP signal at 2500 ng/ml norfloxacin compared to 250 ng/ml (figure 2a and 2b). These findings could explain the observation made by Keren *et al*., (2013) that there was only a small difference in HPF fluorescence between untreated and treated cells at 2500 ng/ml norfloxacin.

It has been observed that bacteriostatic antibiotics such as rifampicin and gentamicin do not induce filamentation or increased cell size [14], which could explain the lack of increase in HPF signal in cells treated with these compounds [1]. In the flow cytometer, fluorescence signals from HPF as well as autofluorescence are measured per event, with large cells being treated the same way as small cells. At the same ROS concentration, large cells will produce higher fluorescence signals than small cells. Vice versa, higher fluorescence signals from larger cells do not necessarily reflect higher ROS concentrations. Hence, flow cytometric analysis of fluorescence signals requires a correction for cell size. Our data suggest that cell size rather than increased ROS production is responsible for the increased fluorescence signal,

Recent studies have questioned the importance of ROS in bactericidal antibiotics lethality. The major arguments include the findings that antibiotics work efficiently under anoxic conditions where neither superoxide nor hydrogen peroxide should be formed. Measurements of the respiration rate under air-saturated growth conditions shows that it is essentially unaffected by exposure to bactericidal antibiotics [8] and that mutants hypersensitive to O_2_^·–^ or H_2_O_2_ exhibit similar sensitivities to bactericidal antibiotics as wild type cells [15], [8]. Although the role of ROS in lethality of bactericidal antibiotics has been much disputed, this research has lead to the important realization that bacterial metabolism at large influences the lethality of antibiotics. Examples of this can be seen with iron-sulfur proteins promoting aminoglycoside mediated killing by enabling their uptake [15]; production of exogenous hydrogen-sulfide protects against several classes of antibiotics [16] and perturbations in TCA cycle activity provides resistance to oxacillin [17]. There is a strong body of evidence documenting the importance of bacterial metabolism for antibiotic susceptibility. However, further research will be needed to dissect if common metabolic pathways or processes augment the lethality of antibiotics.

## Materials and Methods

### Strain and Growth Conditions

The strains used in this study are all derivatives of *Escherichia coli* K-12. The three strains used were wild type *E.coli* MG1655 [18], *E. coli* SAR18 [19] (CSH26 *attB*::bla-P_A1/04/03_-gfpmut3*-T_0_) and *E.coli ftsI2158* (ts) [10]. All strains were grown in 10 ml Luria Bertani broth (LB) (Oxoid, Denmark), incubated at 200 rpm in 100 ml tin foil wrapped narrow-neck Erlenmeyer flasks, at three different temperature settings, 37°C for the majority of the samples and 30°C for *ftsI2158* (ts) mutant before shifted to 42°C at the start of the measurements.

The following antibiotics concentrations were used: 5 μg/ml ampicillin, 5 μg/ml kanamycin and 25 ng/ml, 250 ng/ml or 2500 ng/ml norfloxacin (Sigma Aldrich). The antibiotics were added to the growth medium when the bacteria had reached a cell density of 10^8^ cells/ml. The fluorescent hydroxyphenyl fluorescein probe (3′-*p*-hydroxyphenyl) (Sigma Aldrich) was added one and half hour before the start of the measurements at a concentration of 5 μM.

### Microscopy and Flow Cytometry Analysis

Samples for flow cytometry and microscope analysis were taken before and at the indicated times after addition of antibiotics. The flow cytometry data was recorded with a BD Biosciences Accuri C6 flow cytometer counting 50,000 cells at a flow rate of 35 μl/min or 14 μl/min (data not shown) and a core size of 16-μm or 10-μm. GFP and HPF florescence was excited with a 488 nm argon laser and emission was detected with the FL1 emission filter at 533/30 nm using FL1 photomultiplier tub.

Analysis of the flow cytometry data was performed with Flow Jo X 10.0.6. For microscope analysis an AxioImager Z1 microscope (Carl Zeiss MicroImaging, Inc). The microscope pictures were analyzed with ImageJ Adobe Illustrator and Volocity software (PerkinElmer) software. For statistical analysis, GraphPad Prism version 6.00, GraphPad Software, La Jolla California USA, was used.

## Supporting Information

Figure S1 **Average fluorescent (FL1-A) signal plotted versus average cell size (FSC-A) as measured by flow cytometry for MG1655 cells incubated for three hours with HPF probe, A) without antibiotics, B) 25 ng/ml norfloxacin and C) 250 ng/ml norfloxacin.**
(JPG)

Table S1 **Comparison of absolute and relative cell size between untreated wild type** ***E. coli*** **K-12 (at 0 and 3 hour) and treated cells at 3-hour following exposure of ampicillin (5 ug/ml), norfloxacin (250 ng/ml) and kanamycin (5 ug/ml).** The absolute cell size is given with standard deviation.
(PDF)
