# Supplementary figures and images for: Bactericidal Antibiotics Increase Hydroxyphenyl Fluorescein Signal by Altering Cell Morphology
Source: PLoS One. 2014 Mar 19;9(3):e92231. doi: 10.1371/journal.pone.0092231 (PMC3960231; doi:10.1371/journal.pone.0092231)

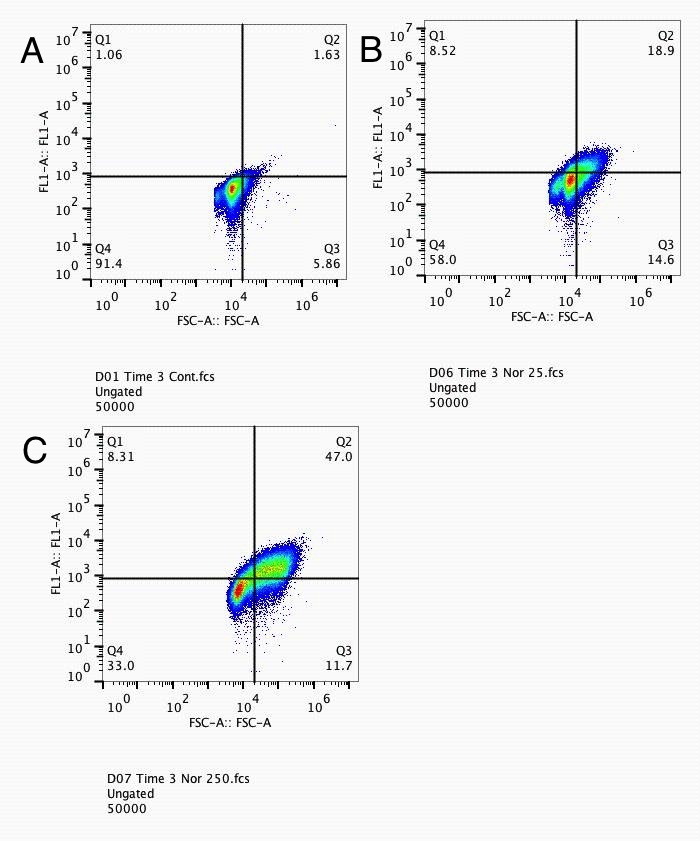

Supplement: Figure S1 — Average fluorescent (FL1-A) signal plotted versus average cell size (FSC-A) as measured by flow cytometry for MG1655 cells incubated for three hours with HPF probe, A) without antibiotics, B) 25 ng/ml norfloxacin and C) 250 ng/ml norfloxacin. (JPG) [file pone.0092231.s001.jpg]
