# Supplementary material for: Bactericidal Antibiotics Increase Hydroxyphenyl Fluorescein Signal by Altering Cell Morphology
Source: PLoS One. 2014 Mar 19;9(3):e92231. doi: 10.1371/journal.pone.0092231 (PMC3960231; doi:10.1371/journal.pone.0092231)
Supplement: Table S1 — Comparison of absolute and relative cell size between untreated wild type E. coli K-12 (at 0 and 3 hour) and treated cells at 3-hour following exposure of ampicillin (5 ug/ml), norfloxacin (250 ng/ml) and kanamycin (5 ug/ml). The absolute cell size is given with standard deviation. (PDF) [file pone.0092231.s002.pdf]

Samples	Average length 3 hours	Relative to wt size
Untreated 0 hour	3.8 µm ± 0.8	1
Ampicillin 3 hours	35.4 µm ± 9.3	9.4
Norfloxacin (250ng/ml) 3 hours	35.6 µm ± 9.8	9.4
Kanamycin 3 hours	6.1 µm ± 2.9	1.6
